# Kainate Receptor-Mediated Modulation of Hippocampal Fast Spiking Interneurons in a Rat Model of Schizophrenia

**DOI:** 10.1371/journal.pone.0032483

**Published:** 2012-03-01

**Authors:** Barbara Gisabella, Vadim Y. Bolshakov, Francine M. Benes

**Affiliations:** 1 Program in Structural and Molecular Neuroscience, McLean Hospital, Belmont, Massachusetts, United States of America; 2 Department of Psychiatry Harvard Medical School, Boston, Massachusetts, United States of America; 3 Program in Neuroscience, Harvard Medical School, Boston, Massachusetts, United States of America; Harvard University, United States of America

## Abstract

Kainate receptor (KAR) subunits are believed to be involved in abnormal GABAergic neurotransmission in the hippocampus (HIPP) in schizophrenia (SZ) and bipolar disorder. Postmortem studies have shown changes in the expression of the GluR5/6 subunits of KARs in the stratum oriens (SO) of sectors CA2/3, where the basolateral amygdala (BLA) sends a robust projection. Previous work using a rat model of SZ demonstrated that BLA activation leads to electrophysiological changes in fast-spiking interneurons in SO of CA2/3. The present study explores KAR modulation of interneurons in CA2/3 in response to BLA activation. Intrinsic firing properties of these interneurons through KAR-mediated activity were measured with patch-clamp recordings from rats that received 15 days of picrotoxin infusion into the BLA. Chronic BLA activation induced changes in the firing properties of CA2/3 interneurons associated with modifications in the function of KARs. Specifically, the responsiveness of these interneurons to activation of KARs was diminished in picrotoxin-treated rats, while the after-hyperpolarization (AHP) amplitude was increased. In addition, we tested blockers of KAR subunits which have been shown to have altered gene expression in SO sector CA2/3 of SZ subjects. The GluR5 antagonist UBP296 further decreased AP frequency and increased AHP amplitude in picrotoxin-treated rats. Application of the GluR6/7 antagonist NS102 suggested that activation of GluR6/7 KARs may be required to maintain the high firing rates in SO interneurons in the presence of KA. Moreover, the GluR6/7 KAR-mediated signaling may be suppressed in PICRO-treated rats. Our findings indicate that glutamatergic activity from the BLA may modulate the firing properties of CA2/3 interneurons through GluR5 and GluR6/7 KARs. These receptors are expressed in GABAergic interneurons and play a key role in the synchronization of gamma oscillations. Modulation of interneuronal activity through KARs in response to amygdala activation may lead to abnormal oscillatory rhythms reported in SZ subjects.

## Introduction

Emotionally stimulated learning and memory in the hippocampus (HIPP) is mediated by glutamatergic afferents from the basolateral amygdala (BLA) [1]. During the generation and retrieval of emotional memories, HIPP activity is synchronized with that of the BLA [2]. Deficits of episodic and associative memory in schizophrenia (SZ) are associated with HIPP dysfunction [3], [4], [5] and may be related to tonic hyperactivation of this brain region [6], [7], [8]. This abnormal output from the HIPP may derive from a loss of GABAergic interneurons [9], [10] and their regulation by the glutamatergic system [11], [12], [13], resulting in a failure of inhibitory activity [14], [15]. When the non-competitive GABA_A_ receptor antagonist picrotoxin (PICRO) is infused into the BLA, a preponderance of abnormalities related to GABA cells has been observed in sectors CA2 and CA3, but not CA1, particularly in the stratum oriens (SO), where fibers from the BLA send a dense projection [16]. By infusing the GABA_A_ antagonist picrotoxin (PICRO) into the BLA, it has been possible to induce changes in GABA cells that are remarkably similar to those seen in postmortem studies [16]. Previous studies have demonstrated that activation of the BLA is associated with a decrease in spontaneous and evoked inhibitory postsynaptic currents (IPSCs) recorded from pyramidal neurons in CA2/3, while changes of this type were not detected in CA1 [17]. Fast-spiking (FS) interneurons showed a dramatic increase in spike frequency in rats receiving PICRO in the BLA [18]. In the same animals, we also observed an increase in currents mediated by hyperpolarization-activated cationic channels (Ih). These channels are known to be involved in the regulation of the excitability of neurons, confirming previous microarray-based gene expression profiling studies showing increased expression of the hyperpolarization-activated Ih (HCN3 and 4) channels in SZs [19], [20]. Gene profiling work has also shown altered expression of KARs in SO sector CA2/3 of SZ subjects [21]. To explore whether KARs mediate the influence of BLA fibers on FS interneurons in SO of CA2/3, we used the rodent model of neural circuitry abnormalities in SZ to examine the cellular mechanisms through which KARs, alone and in combination with hyperpolarization-activated (Ih) channels, may influence the firing rates and other electrical properties of HIPP GABA cells.

## Materials and Methods

### Ethics Statement

Stereotaxic surgery and electrophysiological experiments were performed in HIPP slice preparations. All procedures were approved by the Mclean Hospital IACUC Committee (protocol 10–11/2–20) and conducted in accordance with the National Institutes of Health Guide for the Care and Use of the Laboratory Animals.

### Stereotaxic Surgery

Male P30 Sprague Dawley rats were anesthetized (100 mg/kg ketamine; 40 mg/kg xylazine), and mounted in a stereotaxic apparatus. At postnatal day 30 (P30), a microinfusion system (Alzet mini-osmotic pump [MOP], Model 1002; Cupertino, California) was implanted subcutaneously over the dorsum of the cervical area to chronically deliver PICRO at a concentration of 300 µmol/L in SAL (saline) or in SAL alone (control subjects) was delivered through a microcannula with a diameter of 70–100 µm into the BLA at the following coordinates; B −2.3, L 4.7, V 7.4. Fifteen days later (P45), animals were sacrificed for electrophysiological experiments following continuous infusion of PICRO or SAL. This microinfusion system has been shown to allow more precise targeting of substructures and to minimize trauma at the injection site [22]. The infusion rate of the MOP was reduced from its factory rate of 0.25 µL/hour to 0.125 µL\hour by coating 50% of the pump surface area with paraffin, which resulted in a total volume of 45 µL being infused over a period of 15 days. Immediately after decapitation using a guillotine, the HIPP was removed and placed in a solution containing artificial cerebrospinal fluid (ACSF) and cut into 250–300 µm slices using a vibratome to perform electrophysiological experiments.

### Electrophysiology

The intrinsic firing properties of fast spiking (FS) interneurons in SO of CA2/3 were measured in a whole-cell configuration under current–clamp conditions. Slices (300–350 µm) were continuously superfused with ACSF; no PICRO was present in the bath solution, except in a subset of experiments in which the GABA_A_ antagonist, PICRO was included in the ACSF to test the possible involvement of synaptically-mediated GABA currents on the observed changes in spike frequency.

The ACSF contained (in mmol/L): 119 sodium chloride (NaCl), 2.5 potassium chloride (KCl),2.5 calcium chloride (CaCl_2_), 1.0 magnesium sulfate (MgSO_4_), 1.25 sodium dihydrogen phosphate (NaH_2_PO_4_), 26 sodium bicarbonate (NaHCO_3_), 10 glucose, bubbled with 95% oxygen (O_2_) and 5% carbon dioxide (CO_2_). Recordings were performed under visual guidance (DIC/infrared optics) with an EPC-9 amplifier and Pulse v8.78 software (Heka Elektronik, Lambrecht/Pfalz, Germany). The patch electrodes (3–5 MΩ resistance) contained (in mmol/L): 120 KCl, 5 NaCl, 2 MgCl_2_, 0.1 ethyleneglycol bis-(β-aminoethyl ether)-*N,N′*-tetraacetic acid (EGTA), 10 *N*-2-hydroxyethylpiperazine- *N′* -2-ethane sulfonic acid (HEPES), 4 Mg-adenosine triphosphate (Mg-ATP), 0.3 Na-guanosine triphosphate (GTP) (adjusted to pH 7.2 with potassium hydroxide). All recordings were obtained from cells with somata located within the SO of CA2/3, where anterograde tracing work has previously demonstrated that BLA projections terminate [23]. Interneurons located at the interface between stratum pyramidale (SP) and SO could be distinguished from pyramidal cells on the basis of their morphological appearance. The morphology of the interneurons was analyzed as previously described (see [18]). The resting membrane potential (RMP) was estimated in the absence of injected current, and the input resistance (I/R) was derived from voltage responses to current injection (500 ms; ±100 pA). The analysis of action potentials (APs) included the amplitude, duration (measured at half-amplitude), and half-duration of after-hyperpolarization (AHP) and was performed on first AP discharges elicited by stepwise-increased constant depolarizing current injections at a resting membrane potential. Repetitive firing behavior was assessed during long depolarization current pulses (500 ms) of increasing intensity (50–500 pA) and evaluated by measuring the number of spikes as a function of the amplitude of injected current. The AP discharge frequency was measured at −70 mV between the first two spikes (initial) and at the end of firing (steady state) during a depolarizing current injection (500 ms) at all injected currents tested. Instantaneous firing frequency (IFF), defined as a number of spikes in each 50 ms time interval throughout the depolarizing current step, was calculated from trains of action potentials elicited by single current injections during the first 500 ms of a 1 s current pulse. An agonist of KARs, kainic acid (KA) (200 nM), was applied through the external perfusing solution to observe its effects on slices from picrotoxin treated (PICRO) rats or saline-injected control rats (SAL). To investigate the possible modulatory role of GluR5 and GluR6/7 receptors, the GluR5 antagonist UBP209 (10 µM) or GluR6/7 antagonist NS102 (10 µM) were applied in the experiments on slices from PICRO or SAL injected animals. ZD7288 (10 µM) was applied to block Ih channels. In a separate set of experiments, tertiapin-Q (200 nM) was applied to block G protein-coupled inward-rectifier K^+^ channels (GIRK). All values reported represent the means ± SEM for the number of cells (n) analyzed, with a = number of animals used. Two-tailed Student t test and two-way analysis of variance (ANOVA) with repeated measures were used for statistical comparisons.

## Results

### Intrinsic Membrane Properties of GABAergic Interneurons in SO CA2/3 area of PICRO and SAL Rats in the Presence of Kainic Acid

**Figure 1:**
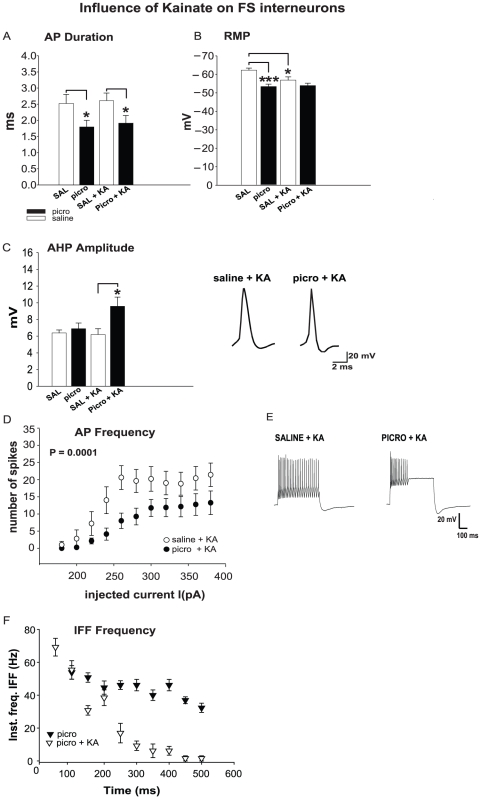
AP firing rate is decreased while AHP is increased in SO of CA2/3 fast–spiking interneurons of PICRO-treated rats in the presence of kainic acid (KA). Application of KA does not affect duration (**A**) or RMP (**B**) in PICRO-treated rats compared to experimental conditions with no drug. In contrast, AHP is increased (**C**) and AP firing rate is decreased in PICRO rats compared with saline–infused animals in the presence of KA (**D, E**). Insets in **C**, **E** show representative traces of interneuron AP responses from SAL (on the left) and PICRO (on the right) infused animals. **F**, Plot of instantaneous firing frequency versus time for spikes elicited by single current steps during the first 500 ms of a 1s current pulse in slices from PICRO rats with or without KA in the external solution. * p = <0.05 or ***p<0.001. Error bars indicate SEM.

As shown previously, PICRO infusion into the BLA increases action potential (AP) frequency in fast-spiking interneurons in SO of CA2/3 area of the hippocampus compared to saline (SAL) controls, accompanied by a decrease of the AP duration and RMP [18]. In the current experiments, we used standard whole-cell patch-clamp recording techniques with the same methods as described in our previous work [18] to examine if changes in electrophysiological properties of interneurons in SO of sector CA2/3 in PICRO infused rats are associated with modifications of KAR-mediated signaling. This was tested by adding kainic acid (KA) to the external solution alone or together with selective blockers of the specific KAR subunits, GluR5 and GluR6/7, which have been shown previously to have altered gene expression in SO sector CA2/3 in SZ subjects [21]. First, we perfused HIPP slices from either PICRO or SAL rats (see Materials and Methods for details) with KA in a concentration which does not induce epilepsy (200 nM). In the presence of KA, the AP frequency of FS interneurons in SO of CA2/3 in PICRO rats was significantly lower compared to slices from SAL control rats (ANOVA: p = 0.0001, n = 10, a = 6 for SAL and n = 13, a = 8 for PICRO rats, Figure 1D), while the magnitude of AHP (mV) was larger in PICRO rats: 6.2±0.7, n = 10, a = 6 for SAL and 9.5±1.1, n = 13, a = 8 for PICRO rats; p = 0.027, (Figure 1C). The input resistance (I/R) of SO interneurons in the CA2/3 hippocampal area in the presence of KA was also higher in slices from PICRO rats (p<0.001) (Table 1). We did not observe an effect of KA on the differences in other intrinsic membrane properties, such as AP duration and RMP (n = 10, a = 6 for SAL and n = 13, a = 8 for PICRO rats). (Figure 1A and 1B) between PICRO and SAL rats. On the other hand, there was a significant decrease in the RMP of interneurons in slices from SAL treated rats after KA application (p = 0.018, Figure 1B). Figure 1F is a plot of instantaneous firing frequency (IFF) versus time for PICRO treated rats before and after KA application during the first 500 ms of a 1 s current pulse. IFF shows a strong AP frequency adaptation in the presence of KA in slices from PICRO-treated rats (ANOVA: p = 0.0001, n = 13, a = 8, before KA application and n = 13, a = 8, after KA application, Figure 1F). These results indicate that the responsiveness of SO interneurons to activation of the KAR signaling pathways might be modulated by the chronic increases of activity in excitatory inputs coming from the BLA to SO of CA2/3 of the HIPP. The observed decreases in the spiking output of SO interneurons in the presence of KA in PICRO rats may, in part, be explained by the increased AHP's between the spikes in the course of firing, resulting in spike frequency accommodation [24], [25], [26], [27].

**Table 1:** I/R values of SO-CA2/3 FS-Interneurons in SAL and PICRO- Treated Rats.

Before KA application	After KA application	After KA+UBP296	After KA+NS102
SAL PICRO	SAL PICRO	SAL PICRO	SAL PICRO
(*n* = 10, a = 6) (*n* = 13, a = 8)	(*n* = 10, a = 6) (*n* = 13, a = 8)	(*n* = 5, a = 3) (*n* = 8, a = 5)	(*n* = 5, a = 3) (n = 5, a = 3)
I/R (MΩ):111.9±12.6 95.7±8.6	I/R (MΩ):128±2.5 252±3.6^b^	I/R (MΩ):125.9±3.3 243.7±4.2^b^	I/R (MΩ):242.4±3.1 229±5.1

After KA+GABA blockers PICRO	After KA+tertiapin-Q PICRO	After KA+ZD7288 PICRO
(*n* = 7, a = 4)	(*n* = 6, a = 3)	(*n* = 6, a = 3)
I/R (MΩ):248.7±3.7	I/R (MΩ):244.9±5.3	I/R (MΩ):224.4±2.9

See Methods sections for details.

SAL, saline; PICRO, picrotoxin; SO, stratum oriens; I/R, input resistance;

a p = 0.015.

b p = 0.001.

### AP Frequency after KA Application in SO Fast Spiking Interneurons of PICRO–Treated Rats is Regulated by GluR5 and GluR6/7 Subunit-Containing Kainate Receptors

**Figure 2:**
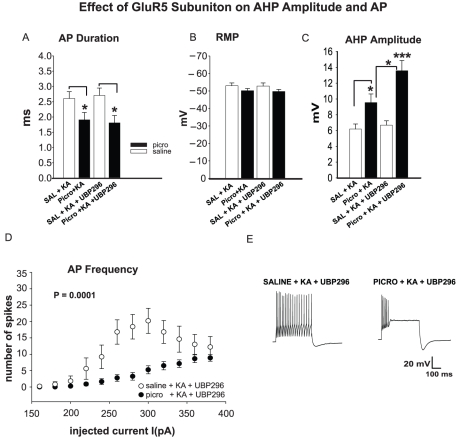
Increased AHP Amplitude and decreased AP firing in the presence of kainic acid is dependent on the GluR5 subunit. **A**, Bath application of UBP296 does not change AP duration and RMP (**B**) of fast-spiking interneurons from PICRO-treated rats compared to experimental conditions with no drug. **C**, **D**, UBP296 application is associated with an increase of AHP and decrease of AP firing rate in SO of CA2/3 fast-spiking interneurons from PICRO-treated rats. A significant increase of AHP amplitude has been observed when comparing SAL+KA+UBP296 versus PICRO+KA+UBP296 groups (p<0.001). UBP296 failed to induce any electrophysiological changes in control rats (**A**–**E**). **E**, This panel shows representative traces recorded before and after application of UBP296 in PICRO versus SAL rats. Results are presented as the mean ± SEM. *p<0.05 or ***p<0.001 versus saline.

**Figure 3:**
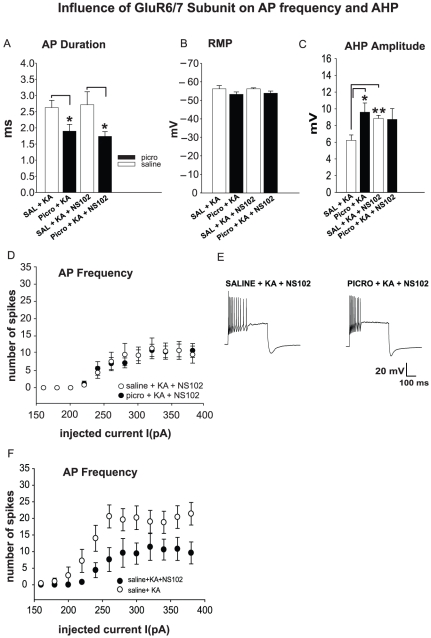
The reduced responsiveness of fast-spiking interneurons from PICRO-treated rats to KA is mediated by GluR6/7 subunit-containing KARs. The GluR6/7 receptor antagonist (NS102) does not have a significant effect on duration (**A**), RMP (**B**), AHP (**C**) or AP frequency (**D, E**) in SO CA2/3 fast-spiking interneurons from PICRO- infused rats compared to conditions with no drug application. **E**, Right side of the panel shows representative traces recorded with and without NS102 in the external solution. **F**, AP spike frequency after NS102 bath perfusion compared to SAL with KA (the plot showing the SAL with KA data is the same as in Figure 1D) (ANOVA: p = 0.0001). *p<0.05 or **p<0.01. Error bars are SEM.

To examine whether GluR5 and/or GluR6/7 KAR subunits are involved in the observed decrease of AP frequency in the presence of KA in SO of CA2/3 of PICRO treated rats, the same experimental conditions described above were employed and HIPP slices were perfused with specific GluR5 and/or GluR6/7 receptor antagonists. In particular, UBP296 (10 µM) was used as a specific GluR5 blocker and NS102 (10 µM) as a combined blocker for the GluR6/7 subunits [28], [29], [30]. Application of UBP296 did not alter AP duration and RMP in SAL versus PICRO compared to the previous estimates obtained without KA application (n = 10, a = 6, before UBP296 and n = 5, a = 3 after UBP296 application for SAL versus n = 13, a = 8, before UBP296 and n = 8, a = 5 after UBP296 application for PICRO rats, Figure 2A, 2B). The application of UBP296, however, led to a significant decrease in AP frequency compared to the PICRO+KA group shown in Fig. 1D (Figure 2D; ANOVA: p = 0.0003, n = 13, a = 8 for PICRO+KA in Figure 1D versus PICRO+KA+UBP296 group, n = 8, a = 5 in Figure 2D) and increase of AHP (9.5±1.1, n = 13, a = 8 for PICRO+KA group in Fig. 1 and 13.63±1.3, n = 8, a = 5 for PICRO+KA+UBP296 group; p = 0.028, Figure 2C). We did not observe differences in the AP firing rates between SAL and PICRO rats in the presence of KA when the antagonist of GluR6/7 subunit-containing KARs NS102 was added to the external solution (Figure 3D, ANOVA: p = 0.95, n = 5, a = 3, for SAL and n = 5, a = 3 for PICRO). The AP duration, RMP and AHP in PICRO-treated rats were also unchanged in the presence of KA and NS102 (Figure 3A–E), while the AHP magnitude was significantly increased in slices from SAL treated rats under the same experimental conditions (p<0.01, Figure 3C). The firing rate of SO interneurons in SAL rats in the presence of KA and NS102 was significantly lower than in slices from SAL rats in the presence of KA only (Figure 3F, ANOVA: p = 0.0001, n = 10, a = 6, before NS102 and n = 5, a = 3 after NS102 application), indicating that KA was increasing the firing output of recorded interneurons through activation of GluR6/7 KARs. Together, the latter observation and the lack of differences in the firing rates between SAL and PICRO rats in the presence of KA and NS102 suggest that activation of GluR6/7 KARs is required to maintain a higher firing rate of SO interneurons in the presence of KA, and that this GluR6/7 KAR-mediated signaling may be diminished in PICRO-treated rats. Thus, apparently, BLA activation may modulate the activity of HIPP FS interneurons through the GluR5, and/or GluR6/7 subunit-containing KARs, resulting in dysfunction in the spike activity, possibly reflecting abnormal interneuronal activity in SZ.

### Decrease of AP Frequency in the Presence of KA is Partially Modulated by GABA_A_ and GABA_B_ Receptors in PICRO-Injected Rats

**Figure 4:**
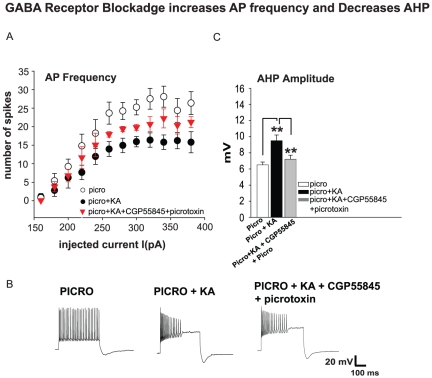
Decrease of AP frequency in the presence of KA in SO CA2/3 fast-spiking interneurons PICRO–treated rats is partially relieved by the blockade of GABA receptors. **A**, The GABA_A_ and GABA_B_ antagonists picrotoxin and CGP55845, respectively, induced a significant increase of AP frequency compared to recordings with KA only application (PICRO+KA plot here is the same as in Figure 1D) (ANOVA: p = 0.05, n = 7) (see red labeled symbol). It also resulted in a decrease in AHP (**C**). **B**, Representative traces recorded in slices from PICRO-treated rats (left) with KA (middle) and CGP55845 with picrotoxin (right) in the external solution. **p<0.01. Error bars are SEM.

To assess whether the GABA system may be directly involved in modulation of AP frequency in the presence of KA in SO of CA2/3 of PICRO treated rats, GABA_B_ (CGP55845, 10 µM) and GABA_A_ (picrotoxin, 50 µM) antagonists were added to the external ACSF. A significant increase of AP frequency was observed in slices from PICRO rats in the presence of KA and GABARs antagonists compared to recordings obtained with KA alone, thus partially rescuing the decreased AP frequency in the presence KA (ANOVA: p = 0.05, n = 13, a = 8 before GABARs antagonists and n = 7, a = 4 after GABARs antagonists application, Figures 4A and 4B). This effect was accompanied by a significant decrease in AHP (9.5±0.7 mV before GABA blockers were applied and 7.2±0.5 in the presence of GABA blockers; n = 7, a = 4, p = 0.002, Figure 4C). There were no changes in any of the other electrophysiological properties. However ANOVA of AP frequency comparing recordings from PICRO rats (with no KA in the bath) and groups with GABARs antagonists in the presence of KA did not show any significant difference (ANOVA: p = 0.114, n = 13, a = 8 before GABARs antagonists and n = 7, a = 4 after GABARs antagonists application).

### Ih Channel blockade Reverses the Increase of AP Frequency Induced by GABA Blockers in SO of CA2/3 Fast-Spiking Interneurons of PICRO-Treated Rats

**Figure 5:**
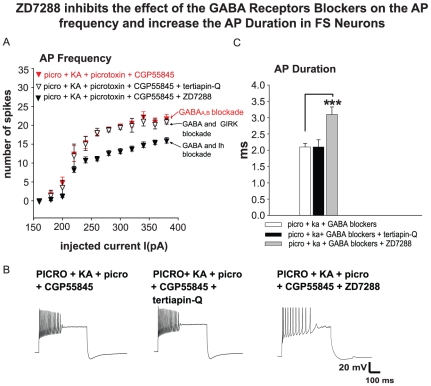
ZD7288 reverses the increase of AP frequency induced by CGP55845 with picrotoxin in SO CA2/3 fast-spiking interneurons PICRO–treated rats. **A**, **B**, Application of the Ih blocker ZD7288 significantly decreases AP frequency and increases duration (**C**) compared to recordings with CGP55845 (GABA_B_ antagonist) and picrotoxin (GABA_A_ antagonist) (the red labeled plot is the same as in Figure 4E) bath application (ANOVA: p = 0.02, n = 6). In contrast, bath perfusion of tertiapin-Q did not have a significant effect on interneuronal electrophysiological properties (**A**, **B**, **C**). Traces on the bottom of the panel were recorded with the application of KA and GABA_A,B_ blockers (left), tertiapin-Q- (middle) and ZD7288 (right). ***p<0.001. Error bars are SEM.

Studies indicating that Ih channels are involved in resetting interneuron firing properties, as well as rhythmic activity regulated through KARs [20], [31], [32] led us to hypothesize that the regulation of AP spike frequency in SO of CA2/3 interneurons from PICRO-treated rats may involve both KARs and GABA receptors, and their functional contributions could be modulated by Ih channels. In a previous study using this model, an increase of Ih amplitude was detected in CA2/3 interneurons from PICRO-treated animals [18]. To test this hypothesis, we added the specific Ih blocker, ZD7288 (10 µM) to the extracellular solution containing KA (and the GABA blockers, CGP55845 to block GABA_B_ and picrotoxin to block GABA_A_ receptors). Under these conditions, ZD7288 reversed the increase of AP frequency induced by CGP55845 and picrotoxin in the presence of KA, resulting in a significant decrease of AP spike frequency (ANOVA: p = 0.02, n = 6, a = 3, Figure 5A and 5B) and increased AP duration (2.1±0.11 ms, n = 12 before ZD7288 and 3.1±0.2, n = 6 after ZD7288; p = 0.0004, Figure 5C). ZD7288 did not affect other electrophysiological properties examined such as AHP amplitude and I/R (see also Table 1). Ih and GIRK channels play complex roles in regulating the amplitude of AHP currents in HIPP neurons [33], [34]. In addition, gene expression profiling work has shown increased expression of the KCNJ3 gene (G protein dependent inwardly rectifying potassium channel; GIRK) in SO CA2/3 of SZ subjects [19].

To determine whether any of the observed electrophysiological changes in interneurons, such as AHP and AP duration and frequency, may also be related to modulation by GIRK channels, we applied tertiapin-Q (200 nM), a blocker of inward-rectifier potassium channels (including GIRK), to the bath in the presence of the GABAR antagonists. Tertiapin-Q application had no significant effect on AP spike frequency (Figure 5A, 5B) or other electrophysiological properties examined. ANOVA analysis showed that interneurons from PICRO injected rats in the presence of KA+GABA blockers+ZD7288 had significantly higher AP frequency compared to interneurons from PICRO injected rats with application of KA only (ANOVA: p = 0.02, n = 19, a = 11). These results demonstrate the involvement of Ih and GABA in observed effects of PICRO treatment possibly mediating the influence of BLA fibers on GABA cells in SO of CA2/3. These results provide insight into the gene expression profiling data from postmortem work [19], indicating that Ih channels may regulate abnormal firing properties of the GABA neurons in SO CA2/3 in SZs in response to increased incoming activity to this sector from the basolateral amygdala.

## Discussion

**Figure 6:**
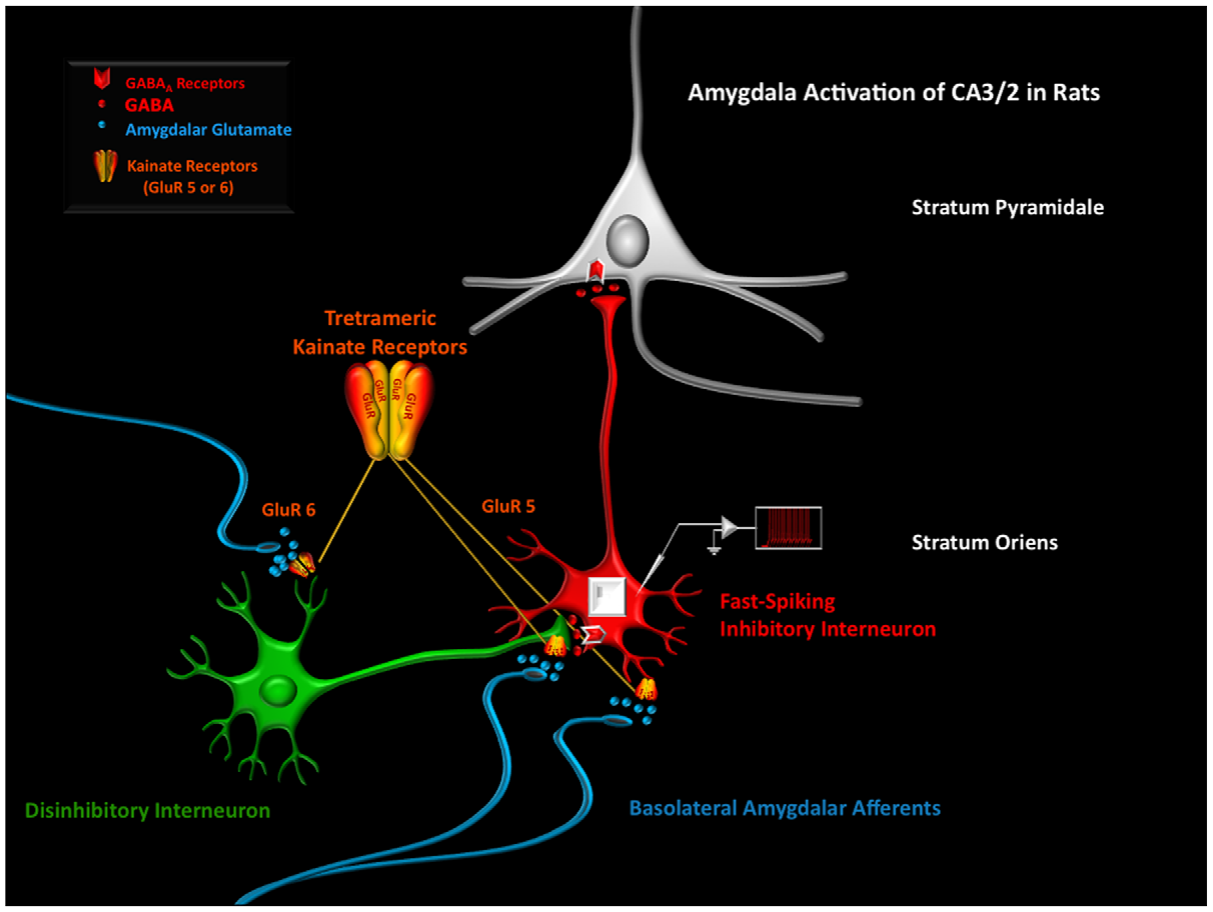
A schematic diagram depicting how an increase of excitatory activity from the BLA might influence the interaction of inhibitory and disinhibitory GABA cells in the SO of CA2/3. The results reported in this study can be best explained by a model in which BLA afferents influence two types of interneurons: one that is a fast–spiking (FS) inhibitory cell (red) and one that is a disinhibitory neuron (green) that forms GABA-to-GABA interactions with the FS interneuron (**1**). The diagram suggests that BLA fibers may provide two different KARs-mediated glutamatergic interactions with the disinhibitory neuron. Because PICRO-infused rats showed a significant increase in the amplitude of AHPs in FS-cells, these glutamatergic fibers probably stimulate the KARs located in dendrites of disinhibitory neuron through axodendritic connections (**2**). Additionally, the further increase in the amplitude of AHPs recorded in FS cells observed in PICRO-treated rats with blockade of the GluR5 subunits of KARs suggests that BLA fibers may also provide a pre-synaptic inhibitory effect of the dysinhibitory axon terminal synapting on the FS cells. This last effect is mediated by GluR5 or 6/7 on GABA-to-GABA terminals (**3**). BLA fibers have been found to form axo-axonic connections in cortical neuropil (Cunningham et al. 2002) and, in the SO of CA2/3, similar connections with the axon terminations of disinhibitory interneurons maybe present. This circuitry model provides new insights as to how BLA fibers may contribute to the synchronization of oscillatory rhythms generated in the amygdala and hippocampus during normal and abnormal cognitive states.

The results reported here suggest that the activation of amygdalar projections to the SO of CA2/3 is mediated, at least in part, by KARs and unique changes in the electrical properties of FS interneurons. These results are consistent with the idea that KARs are expressed by GABAergic interneurons [35] and may play a significant role in mediating the influence of the BLA on HIPP activity in SZ and BD [21]. KARs can reset interneuron firing through small depolarizing currents that produce profound changes in neuronal excitability [31]. The decreased ability of KA to maintain the AP spike frequency in PICRO-treated rats, the effects of the GluR5 as well as GluR6/7 antagonists, point to the cellular and molecular complexity of this mechanism. The reduction of excitability and increased accommodation is likely due in part to the observed change in AHP (see fig. 1). Evidence for involvement of AHP in action potential frequency adaptation includes studies of the rat hippocampus, where blockade of the AHP conductance led to reduction in adaptation [36], and similar effects in other studies [24], [25], [27]. KARs play an important role in glutamatergic transmission, one that can be remarkably cell-type specific [37], [38]. Interneurons mainly express two distinct KAR subtypes: one that predominantly contains the GluR5 subunit and another that contains both the GluR6 and 7 subunits [39], [40], [41]. A key function of the GluR5 subunit in interneurons is the control of GABA release and the resultant regulation of tonic inhibition in pyramidal cells with which they are inter-connected [40]. During development, the GluR5 subunit plays an important role in promoting activity dependent maturation of HIPP circuitry by inhibiting glutamate release; as this circuitry reaches maturity, the inhibition of glutamate release is drastically reduced [40], [42]. BLA activation of FS neurons in the SO of CA3/2 of PICRO-infused rats is associated with a marked increase in firing rate and a prolonged activation of FS neurons [18], suggesting that this fiber system may directly stimulate these inhibitory interneurons (Fig. 6 left).

It is reasonable to assume that KARs are stimulated by the addition of KA to the medium in a hippocampal slice preparation. In rats receiving PICRO infusions in the BLA, however, the firing rate of FS neurons shows a paradoxical decrease in the responsiveness to KA application. This effect is associated with a simultaneous increase in the amplitude of AHPs recorded from the same cells. Under these experimental conditions, the associated decrease of spike frequency in FS cells could be partially related to an increase of accommodation due to the observed change of AHP, but possibly also to an activation of a slow subthreshold conductance, one that could either lower the excitability of FS cells or diminish their AP propagation [43]. As shown in Figure 6, an alternative mechanism that could help to explain a reduction of AP spike frequency in FS cells of PICRO-infused rats invokes the presence of a second population of GABA cells, one that is disinhibitory in nature and involved in mediating the effect of KAR activity by amygdala fibers. A GABA-to-GABA mechanism has been suggested in schizophrenia by postmortem studies showing a decrease of interneurons in sectors CA2 [9], particularly those containing parvalbumin [44], [45]; a compensatory upregulation of GABA_A_ receptor binding activity was also detected on interneurons in CA2/3, but not pyramidal cells [46]. These results have suggested that GABA-to-GABA disinhibitory activity may play an important role in the modulation of microcircuitry in SO of sectors CA2/3. If a mechanism of this type is diminished in SZ, it could contribute to disturbances of rhythmic activity generated at this site by GABA cell networks. In the PICRO infused rats described in this report, the increase of AHP amplitude recorded in FS cells in the presence of kainic acid (Fig. 6 right) suggests that BLA fibers may stimulate disinhibitory neurons via GluR5 or 6/7-containing KARs [40], [43] and, in this way, inhibit the firing of inhibitory FS neurons. A mechanism of this type could be involved in SZ and BD, particularly since the expression of the GluR5 subunit is significantly reduced at this locus in both disorders [19].

BLA fibers release glutamate and probably stimulate KARs located on the dendrites of both inhibitory and disinhibitory neurons (Figure 6). The disinhibitory cell probably releases GABA onto the inhibitory FS cell via a classic synaptic mechanism, as the addition of antagonists of GABA-A and GABA-B receptors prevent the increase in the amplitude of AHPs recorded in these cells in PICRO-infused rats [18]. Blocking the excitation of a disinhibitory cell might be expected to reduce its influence on a post-synaptic FS neuron and result in an either an increase or perhaps no change in its AP firing rate. In PICRO-infused rats, however, blockade of the GluR5 subunit by UBP296 further decreases AP firing rate in FS cells, and also increase the amplitude of AHPs. This response may be pointing in part to a presynaptic inhibitory mechanism that also influences GABA release via GluR5-containing KARs [47], [48], [49]. In the PICRO-infused rats, blockade of such a mechanism could also induce changes in AHPs in the FS cells, which in turn may result in the observed reduced excitability and increase of accommodation. However, the observed decrease of the firing rate in the presence of NS102 in slices from SAL rats with KA in the external medium suggests that the PICRO treatment-induced suppression of sensitivity of SO interneurons to KA may also be mediated by GluR6/7 subunit-containing KARs through a complex mechanism which requires further study. The observed results that interneurons from PICRO injected rats in the presence of KA+GABA blockers+ZD7288 had significantly higher AP frequency compared to application of KA only also suggest the involvement of Ih and GABA in the KA effects mediating the influence of BLA fibers on GABA cells in SO of CA2/3. It is possible however that the increased AP frequency may be related to a number of mechanisms that can potentially include accommodation, as suggested above, or depolarization block [50]. Future experiments will focus on identifying the mechanism behind this observed increase in AP frequency.

The HIPP is composed of diverse subpopulations of GABAergic interneurons [51] and it is likely that a complex interplay of inhibitory and disinhibitory neurons contributes to the coordination of rhythmicity within local circuits of the trisynaptic pathway [52]. In psychotic disorders, increased activation of the BLA may alter the firing of inhibitory interneurons in the SO of CA2/3, possibly through a combination of pre- and post-synaptic mechanisms mediated by GluR5 and 6/7-containing KARs expressed by disinhibitory neurons.
